# Spatial and Temporal Changes in *Taenia solium* Cysticercosis Seroprevalence among Pigs in the Southern Highlands of Tanzania

**DOI:** 10.1155/2024/7261324

**Published:** 2024-03-06

**Authors:** Mwemezi L. Kabululu, Bamidele N. Ogunro, Helena A. Ngowi

**Affiliations:** ^1^Tanzania Livestock Research Institute (TALIRI)-Mpwapwa, Dodoma, Tanzania; ^2^University of Ibadan, Ibadan, Nigeria; ^3^Sokoine University of Agriculture (SUA), Morogoro, Tanzania

## Abstract

*Taenia solium* remains among the major causes of epilepsy in sub-Saharan Africa. Understanding the geospatial distribution of the parasite is important to inform control strategies. This study assessed spatiotemporal changes in the *T. solium* infection among pigs from a rural area in southwestern Tanzania. Secondary data from a previous repeated cross-sectional study in the Mbeya and Mbozi districts were used, involving sixteen villages, eight from each of the two districts. Serum samples were collected from 482, 460, and 421 pigs in 221, 196, and 139 households, respectively, at seven-month intervals. Ag-ELISA was used to analyse the samples for circulating antigens. Geographic coordinates of the pig corrals adjacent to the households were also recorded using a hand-held GPS device. SatScan v9.7 software was used to perform purely spatial scan analysis, based on the Bernoulli distribution model. Spatial clustering was assumed based on the relative risk of *T. solium* seropositivity. Statistical significance (set at 0.05) of the cluster was determined by comparing the log-likelihood ratio with the null distribution derived by Monte Carlo simulations. The window with the highest log-likelihood ratio was regarded as a primary cluster. Results showed significant clustering of *T. solium* cases with the presence of a single primary cluster during each phase. The relative risk of *T. solium* infection among pigs within the primary cluster areas ranged from 3.0 to 6.7. The radii of the clusters expanded from 1.83 to 27.7 km before shrinking to 1.5 km, and cluster location drifted from north to central and then to the southern part of the study area. These findings suggest that with restricted resources, control measures in the area could be focused on pockets of high transmission. However, changes in the size and location of clusters suggest that to be effective, interventions should be instituted soon after clusters are identified.

## 1. Introduction

Global demand for meat is soaring as a result of population growth and income rise [1]. Among livestock, pigs have a greater potential of meeting this demand due to their high fecundity, early maturity, high growth rate, short generation interval, high feed conversion efficiency, and relatively small space requirement. The pig sector in Tanzania has been growing markedly, being an important source of animal protein and decent incomes both in rural and urban areas. However, among the challenges faced by the sector is *Taenia solium*, a pork tapeworm responsible for serious health problems and economic losses. The parasite can be transmitted from pigs to humans, leading to a disease called taeniasis, or from humans to pigs, causing porcine cysticercosis. Inadvertently, transmission can occur between humans leading to human cysticercosis.

Taeniasis (infection by the adult tapeworms) results from ingestion of larval forms of the parasites (cysticerci) which may be present in undercooked or raw pork. This is common where home slaughter of pigs is common and pork inspection is lacking or is insufficient leading to the consumption of infected pork [2]. People with taeniasis (tapeworm carriers) pose a significant public health risk as they may shed (with faeces) thousands of infective eggs into the environment, leading to substantial contamination, particularly in areas where “open defaecation” is common and personal hygiene is poor.

Cysticercosis, on the other hand, is an infection with the cysticerci, and it is brought on by ingesting viable parasite eggs expelled by tapeworm carriers. In humans, cysticercosis results from the consumption of food or water tainted with viable parasite eggs [3]. Human cysticercosis is responsible for a range of clinical manifestations, most important being epilepsy resulting from cysticercosis of the neural tissues (neurocysticercosis: NCC). *T. solium* is listed as one of the major causes of acquired epilepsy, particularly in areas where the parasite is endemic and is reckoned as the most significant foodborne parasite in the world [4]. The World Health Organisation has included both cysticercosis and taeniasis on its list of neglected tropical diseases [4].

Porcine cysticercosis follows pigs' access to human waste. It is, therefore, facilitated by the lack, infrequent, or improper use of latrines [5, 6] and free-ranging pigs [7, 8]. Apart from having public health implications by being the cause of taeniasis, porcine cysticercosis also has substantial economic consequences, particularly in regions where pig farming plays a crucial role in livelihoods and food security. During a meat inspection, the presence of cysticerci in the muscular tissues causes infected carcases to be rejected. This not only results in direct financial losses for pig farmers but also affects the entire value chain, including traders, processors, and retailers [9, 10].

The factors facilitating the transmission of *T. solium* are consistent with poor living conditions and standards. For instance, because of the significance of faecal contamination in transmission, *Taenia* spp. infections are frequent in low-income countries with poor sanitation. Thus, despite the parasite's global distribution, it is most prevalent in Africa [11, 12], Latin America [13, 14], and in south and south-east Asia [15, 16].

Understanding the distributional patterns of *T. solium* infections is crucial to inform control strategies. The complex interactions between humans, pigs, and the environment create localised pockets of transmission, resulting in the possible formation of spatial disease clusters. Several studies have documented the clustering of infected pigs and humans. For example, studies by Lescano et al. [17] and Pray et al. [18] showed cases of porcine cysticercosis tended to cluster around homes that had tapeworm carriers; while Pray et al. [18] reported a clustering of cases of taeniasis cases around infected pigs. A study in the Mbulu district of northern Tanzania found local clusters of porcine cysticercosis prevalence and incidence [19].

The presence and distribution of pig infection are a reliable indicator of active transmission of *T. solium* [20]. This is due to pig's significantly shorter life span and the fact that they typically do not move across great distances [21]. Hence, establishing disease clusters enables the identification of high-risk areas (hotspots), thereby enabling targeted surveillance and control efforts. By focusing interventions on areas with the highest disease transmission and burden, resources can be optimized, leading to more effective control strategies.

This study was conducted to determine the geographic distribution of pigs seropositive for *T. solium* cysticercosis in the rural districts of Mbeya and Mbozi, within the southern highlands of Tanzania. The study also intended to elucidate whether the clustering changed over time.

The results of this study are anticipated to advance our understanding of the parasite's epidemiology in endemic regions and inform us of effective and sustainable control measures.

## 2. Methodology

### 2.1. Study Area

Data used in this study were collected from 221 households in rural districts of Mbeya and Mbozi in Mbeya and Songwe regions, respectively, in the southwestern highlands of Tanzania. The districts are situated between latitudes 8°14′ and 9°24′S and longitudes 32°04′ and 33°49′E. The districts experience two major climatic seasons; the rainy season which lasts from November to May and the dry season which extends from June to October. Regions in the southwestern highlands of Tanzania are the major pig production area in the country, with more than half of the country's pig population [22].

The two districts are mostly rural, and pig rearing is among the important income-generation activities. Many households rely on pig farming as a means of diversifying their income sources, thereby reducing their vulnerability to economic shocks and agricultural uncertainties. Pig rearing in the area was to a greater extent on a smallholder scale with the majority of farmers owning one to five pigs [23]. Transmission of *T. solium* was assumed to be local as sourcing of replacement and/or fattening stocks was almost exclusively from neighbouring households within a locality [23]. A number of previous studies have identified the two districts to be *T. solium* transmission hotspots [7, 24, 25].

### 2.2. Data Collection

Data used in this study were secondary data from a repeated cross-sectional study group randomization study which assessed an integrated intervention consisting of improvement in pig housing, feeds and feeding practices, as well as strategic use of oxfendazole to treat pigs [26]. Sixteen villages were included in the study, eight from each of the two districts; with 10 villages receiving the intervention villages and six serving as controls. To assess the infection status of pigs, serum samples were taken from 482, 460, and 421 pigs in 221, 196, and 139 households at seven-month intervals. Sampled pigs were older than three months and excluded pregnant or nursing sows. The first round of data was collected during the start of the dry season (June-July), the second round during the following rainy season (January-February), and the third round during the next dry season (August-September). The samples were analysed for circulating antigens using monoclonal-based sandwich Ag-ELISA as initially described by Brandt et al. [27] and later modified by Dorny et al. [28] and Sikasunge et al. [29]. In addition, the geographic coordinates of the pig corrals adjacent to the households were recorded using a hand-held GPS receiver.

Statistical analysis failed to detect any significant effect of the intervention on the disease prevalence [26]. Hence, all 16 villages were included in this mapping study.

### 2.3. Data Analysis

Geographical coordinates of households with infected pigs were extracted. Maps were created using QGIS version 3.24.1-Tisler. SatScan v9.7 (https://www.satscan.org) geostatistical software was used to perform purely spatial scan analysis [30] on *T. solium* test results. The scan was done separately for each of the three phases of the screening. The scan was for high rate clusters of *T. solium* seropositive pigs using the Bernoulli distribution model (a test model based on the case and control, in which the cases were positive pigs, while the controls were negative pigs). A circular window representing a potential area of *T. solium* was used for the scan.

The null hypothesis of spatial clustering presupposes that the relative risk of *T. solium* seropositivity is the same within the window weighed against without, which nullifies the effects of covariants and eliminates sampling bias. Spatial dimensions of the scan windows were restricted to 50% of the total population of sampled pigs. A cluster was set to contain at least 10% of the positive cases. Statistical significance (set at 0.05) of the cluster was determined by comparing the log-likelihood ratio with the null distribution derived by Monte Carlo simulation set at 999 replications. The window with the highest log-likelihood ratio was regarded as the cluster most unlikely to be due to chance (i.e., primary cluster). Other clusters were regarded as secondary.

## 3. Results

**Figure 1:**
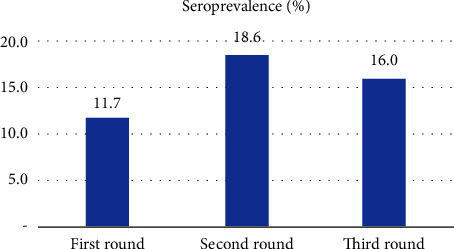
Seroprevalence of *Taenia solium* cysticercosis collected from 482, 460, and 421 pigs at seven-month intervals in Mbeya (rural) and Mbozi districts, Tanzania.

*Taenia solium* seroprevalence increased from 11.7% (55/472) in the first phase to 18.6% (85/457) in the second phase and dropped to 16.0% (64/401) in the third phase (Figure 1).

There was significant clustering of positive cases characterised by the presence of only a single significant cluster of cases during each phase of the study within the two study districts. The relative risk of *T. solium* infection among pigs within the primary cluster areas ranged from 3.0 to 6.7 and the *p* values ranged from 0.001 to 0.024. The location and radii of the clusters changed over the course of the study.

**Figure 2:**
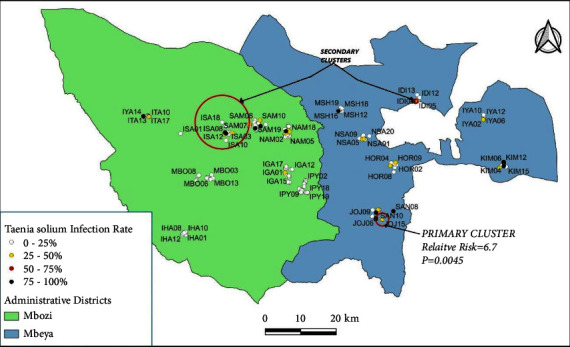
A purely spatial scan for high rates of *Taenia solium* among household pigs in Mbeya (rural) and Mbozi districts of Tanzania during the first phase of data collection. One primary and two secondary clusters were identified.

During the first phase of the study, the relative risk of infection within the identified primary cluster was 6.7 (*p* = 0.0045). The identified primary cluster was small (radius = 1.83 km) and contained only five pig-keeping households within Jojo village in Mbeya District (cluster centroid: 9.136900 S, 33.378520 E) (Figure 2). There were also two secondary clusters; one bigger and the other smaller than the primary cluster.

**Figure 3:**
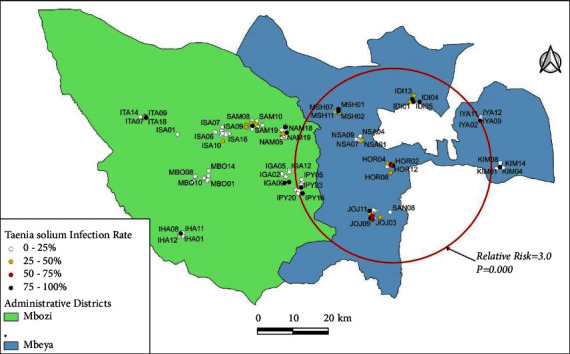
A purely spatial scan for high rates of *Taenia solium* among household pigs in Mbeya (rural) and Mbozi districts of Tanzania during the second phase of data collection. One large primary was identified.

In the second phase, the relative risk was 3.0 (*p* = 0.0001), and a dramatic increase in the cluster size was observed (radius = 27.7 km; centroid: 8.996000 S, 33.414600 E) which now contained 74 pig-keeping households spread across 8 villages (Figure 3) (Horongo, Idimi, Iyawaya, Jojo, Mshewe, Nsalala, and Santilya in Mbeya District; and Ipyana in Mbozi District).

**Figure 4:**
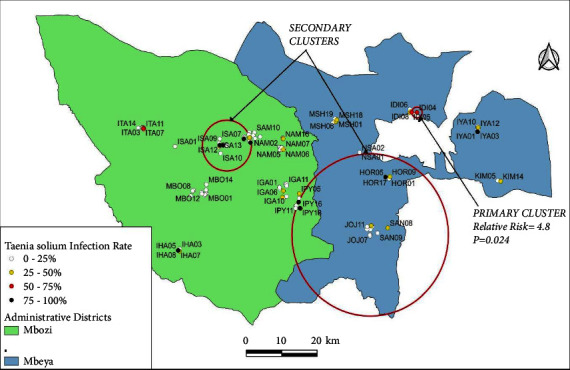
A purely spatial scan for high rates of *Taenia solium* among household pigs in Mbeya (rural) and Mbozi districts of Tanzania during the third phase of data collection. One primary and two secondary clusters were identified.

The relative risk of infection in the third phase was 4.8 (*p*=0.0024), and the cluster size was reduced dramatically (radius = 1.46 km), containing only two pig-keeping households within Idimi village in Mbeya District (cluster centroid: 8.832800 S, 33.482200 E) (Figure 4). There were also two secondary clusters which were bigger than the primary cluster.

In addition, the cluster of porcine *T. solium* cases drifted from south to central and then to the northern part of the Mbeya District, expanding significantly during the second phase to include a few cases within the fringes of the Mbozi District and then shrinking in the third phase of the survey.

## 4. Discussion

This study describes, for the first time, the spatial and temporal distribution of *T. solium* cysticercosis in pigs from the main pig production area in Tanzania. These findings demonstrated that the infection was heterogeneously distributed over the study area, as clusters of infected households were identified, which indicated pockets of *T. solium* hyperendemicity. According to these findings and those of earlier studies, transmission from human to pig is highly focal and depends on local environmental contamination by tapeworm carriers [17].

To our knowledge, only one study in Tanzania had assessed spatial clustering of *T*. *solium* cysticercosis cases in pigs. Ngowi et al. [19] established an overall significant clustering of porcine cysticercosis in Mbulu District of northern Tanzania and detected significant local clusters of the infection. An earlier study in the area had discovered village-level clustering of infection in pigs by using a Bayesian analysis [6].

Although this present study did not directly assess factors that could be responsible for the observed clustering, the distribution is likely a result of an interplay among socioeconomic, bahavioural, and environmental factors in infection risks among households and pigs [31]. Proximity to a tapeworm carrier has been determined as the primary risk factor for the occurrence of *T. solium* cysticercosis in pigs [32]. Therefore, the clustering of pig infection could be attributed to the dynamics of interaction between pigs and tapeworm carriers. A previous study in the area showed that a history of porcine cysticercosis in a household and rearing pigs sourced from the same household were among the risk factors for infection. This reflected clustering of infection, denoting the presence of factors within households, which enhanced the *T. solium* transmission [7]. Although this previous study did not determine infection in humans, the presence of tapeworm carriers could probably be one of such factors within households.

Elsewhere, previous studies have reported a gradient of pig infection towards the location of tapeworm carriers [17, 18]. In Peru, Pray et al. [18] using data from postmortem examination of pigs reported a geographical correlation between tapeworm carriers and infected pigs. It was also found that the prevalence of pig infection was highest within a perimeter of 50 meters surrounding a tapeworm carrier and decreased monotonically as distance increased. Also, in Peru, using necropsy-confirmed cysticercosis data, Lescano et al. [17] found that seven out of ten (70%) pigs reared by tapeworm carriers were infected, and this was significantly higher compared to 11% and 0.5% for pigs living <500 and >500 m from a tapeworm carrier. Therefore, it is highly probable that the clusters identified in the present study are areas that surrounded tapeworm carriers and such areas should be targeted for further surveillance and/or intervention.

Another important finding of this study is the change over time in size (and drifting) of the identified clusters. This study has demonstrated the expansion and shrinking of the clusters, which seems to mimic the increasing (from 11.7% to 18.6%) and decreasing (from 18.6% to 16%) of the prevalence of the disease. The prevalence increased from the dry season to the next rainy season and then dropped from the rainy season to the next dry season. Earlier studies in the area demonstrated temporal fluctuations in the seroprevalence of the porcine cysticercosis [25, 26]. The fluctuations were attributed to the seasonal differences in the movement of pigs and hence transmission dynamics of the parasite. Pig movements and feeding behaviour seem to play a pivotal role in the distribution and transmission of the parasite. Variations in movements of most species in terms of distance covered and areas of displacement are influenced by intrinsic and extrinsic factors, which may include weather-related variables, terrain (such as the presence of water bodies and crop fields), and individual characteristics [33].

The reported temporal fluctuations in the prevalence of pig infection in the study area are consistent with a practice of corralling pigs during the period of planting and growing crops in the fields (rainy season) and letting them loose after harvesting (dry season). Hence, it may be assumed that the dry season is the period when more pigs are exposed to infection risk from environmental contamination with a higher transmission and infection rate, resulting in a higher prevalence detected during the next rainy season. On the contrary, due to a higher rate of corralling pigs during the rainy (cropping) season, the rate of transmission is reduced with the observed lower prevalence during the next dry season.

Seasonal pig confinement has also been reported in Kenya [34, 35] and in Burkina Faso [36], and similar reasons were cited. Elsewhere, other studies have reported seasonal differences in pig movements and rates of transmission risks, with different patterns and reasons cited. For example, in Peru, Pray et al. [37] reported more pig roaming during the rainy season with home ranges 61% larger compared to the dry season. The pattern was associated with an increase in the availability of wild fruits, foliage, and natural streams for pigs to bathe in during the rainy season. Studies in Mexico by Martínez Pérez et al. [38] and Copado et al. [39] discovered that pigs traveled roughly twice as far in the rainy season as they did in the dry season, and this was attributed to availability of more feed in the rainy season. In addition, during the dry season, temperatures are higher (up to 32° C) which is out of the comfort zone of pigs, hence pigs prefer to stay in the shade resting and exploring rather than walking and feeding.

Identification of these clusters could help to make informed decisions such as studying the influence of potential risk factors and transmission pathways or instituting focused intervention measures. O'Neil et al. [40] demonstrated in a 2-year cluster randomized trial in Peru that the ring strategy (ring screening and ring treatment) and mass drug administration (MDA) equally reduced *T. solium* transmission in the area. However, the ring strategy achieved control by treating fewer people (within the transmission hotspots) than in MDA, which ignores clustering. Only 19.3% of the people received niclosamide through the ring strategy compared to 88.5% who received the treatment through MDA.

Hence, the findings of the current study provide prospects for using ring strategies for the control of the parasite in Tanzania. However, changes in the size and location of clusters suggest that time is of the essence and so, to be effective, interventions should be instituted soon after clusters are identified. A drawback of the ring strategy is the operational complexity involved because of the need for surveillance to detect infected pigs and hence identify areas to focus intervention efforts. Hence, there is a need to strengthen surveillance systems which should involve more community surveillance.

A limitation of this study worth noting is that it did not determine other factors that could be related to the observed distribution of diseased pigs. A study in the Democratic Republic of Congo that assessed the correlation between taeniasis and porcine cysticercosis found no overlap between significant clusters of tapeworm carriers and infected pigs [41]. It was determined that the distribution of infected pigs alone cannot be used to determine the proper focus for the ring strategy. Hence, the results of this study should be interpreted with caution, and further studies are warranted to assess other possible factors associated with the observed clustering of infected pigs and changes in the size and location of the clusters.

## 5. Conclusion

In conclusion, this study demonstrated the presence of clusters of *T. solium* cysticercosis in pigs from an endemic area in the southwestern highlands of Tanzania. These findings suggest that to be cost-effective, control measures should be targeted to these pockets of high infection rates. However, changes in the size and location of clusters indicate that to be effective, interventions should be time sensitive and should be instituted soon after clusters are identified.

## Data Availability

The data used to support the findings of this study are available from the corresponding author upon a reasonable request.
